# Supplementary material for: Annexin A1 Expression Is Associated with Epithelial–Mesenchymal Transition (EMT), Cell Proliferation, Prognosis, and Drug Response in Pancreatic Cancer
Source: Cells. 2021 Mar 15;10(3):653. doi: 10.3390/cells10030653 (PMC8000658; doi:10.3390/cells10030653)
Supplement: Supplementary file 1 [file cells-10-00653-s001.pdf]

Supplemental Materials

Oshi et al. "Annexin A1 Expression is Associated with Epithelial-Mesenchymal Transition (EMT), Cell Proliferation, Prognosis, and Drug Response in Pancreatic Cancer"

1. Table S1: Correlation between *ANXA1* expression and EMT-associated genes in the hallmark EMT gene sets in the TCGA cohort.
2. Table S2: Clinical and pathological features between *ANXA1* low and *ANXA1* high pancreatic cancer in the TCGA cohort.
3. Table S3: Pancreatic cancer cell lines.
4. Figure S1: Histogram of *ANXA1* expression in the TCGA and GSE57495 cohorts.
5. Figure S2: Association of *ANXA1* with epithelial-mesenchymal transition (EMT)-associated genes in the TCGA and GSE57495 cohorts.

Table S1. Correlation between *ANXA1* expression and EMT-associated genes in the hallmark EMT gene sets in the TCGA cohort.

r	*P*	Genes
0.655	<0.01	ITGA5
0.642	<0.01	AREG
0.61	<0.01	TGM2
0.58	<0.01	ITGA2
0.573	<0.01	CALU
0.567	<0.01	LAMC2
0.563	<0.01	MMP14
0.556	<0.01	TGFBI
0.553	<0.01	SERPINH1
0.551	<0.01	PLOD1
0.55	<0.01	NT5E
0.549	<0.01	PLOD2
0.544	<0.01	VCAN
0.543	<0.01	ITGB5
0.543	<0.01	FLNA
0.541	<0.01	BMP1
0.54	<0.01	ITGB1
0.538	<0.01	LOXL2
0.536	<0.01	SERPINE1
0.536	<0.01	EMP3
0.532	<0.01	CD44
0.53	<0.01	LOX
0.527	<0.01	SNAI2
0.525	<0.01	FSTL1
0.524	<0.01	BDNF
0.523	<0.01	PMP22
0.52	<0.01	ITGAV
0.517	<0.01	COL5A1
0.517	<0.01	INHBA
0.51	<0.01	GREM1
0.505	<0.01	COL5A2
0.504	<0.01	SPOCK1
0.504	<0.01	PLAUR
0.499	<0.01	SDC4
0.498	<0.01	P3H1
0.495	<0.01	COL3A1
0.493	<0.01	CDH11
0.493	<0.01	DKK1
0.492	<0.01	COL1A1
0.489	<0.01	POSTN
0.488	<0.01	FN1
0.488	<0.01	TNC
0.486	<0.01	LAMA3
0.485	<0.01	SPARC
0.484	<0.01	COL6A3
0.484	<0.01	THBS1
0.478	<0.01	COL7A1
0.477	<0.01	FAP
0.476	<0.01	GPC1

0.476	<0.01	TNFRSF12A
0.475	<0.01	TPM4
0.474	<0.01	COL12A1
0.473	<0.01	THBS2
0.472	<0.01	MMP2
0.472	<0.01	GEM
0.472	<0.01	PDGFRB
0.468	<0.01	LAMC1
0.468	<0.01	LGALS1
0.467	<0.01	NTM
0.466	<0.01	FBN1
0.466	<0.01	ITGB3
0.465	<0.01	COL1A2
0.464	<0.01	COL4A1
0.46	<0.01	COL5A3
0.457	<0.01	FSTL3
0.455	<0.01	WNT5A
0.454	<0.01	CTHRC1
0.45	<0.01	GADD45A
0.45	<0.01	MXRA5
0.449	<0.01	NID2
0.447	<0.01	CALD1
0.446	<0.01	COL4A2
0.444	<0.01	ACTA2
0.438	<0.01	LUM
0.433	<0.01	ADAM12
0.432	<0.01	PTHLH
0.431	<0.01	EFEMP2
0.431	<0.01	GLIPR1
0.428	<0.01	GJA1
0.427	<0.01	DAB2
0.426	<0.01	TPM1
0.426	<0.01	PMEPA1
0.426	<0.01	NOTCH2
0.425	<0.01	PRRX1
0.425	<0.01	GAS1
0.423	<0.01	BASP1
0.423	<0.01	FERMT2
0.42	<0.01	IGFBP3
0.418	<0.01	DST
0.418	<0.01	MFAP5
0.415	<0.01	LRP1
0.414	<0.01	VIM
0.413	<0.01	DCN
0.413	<0.01	DPYSL3
0.412	<0.01	PCOLCE
0.412	<0.01	CD59
0.407	<0.01	TIMP3
0.405	<0.01	FBLN2
0.402	<0.01	COL6A2
0.4	<0.01	VEGFC
0.393	<0.01	COPA
0.392	<0.01	WIPF1

0.39	<0.01	THY1
0.39	<0.01	FGF2
0.388	<0.01	OXTR
0.387	<0.01	BGN
0.387	<0.01	GPX7
0.385	<0.01	COL11A1
0.381	<0.01	HTRA1
0.379	<0.01	COL8A2
0.375	<0.01	FBLN1
0.374	<0.01	MYLK
0.366	<0.01	TAGLN
0.364	<0.01	CXCL8
0.362	<0.01	COLGALT1
0.361	<0.01	COL16A1
0.359	<0.01	TNFAIP3
0.358	<0.01	MYL9
0.353	<0.01	SDC1
0.353	<0.01	IL15
0.352	<0.01	PDLIM4
0.351	<0.01	TGFB1
0.351	<0.01	ECM2
0.35	<0.01	LOXL1
0.347	<0.01	LAMA2
0.347	<0.01	SAT1
0.335	<0.01	PPIB
0.335	<0.01	CRLF1
0.334	<0.01	TPM2
0.333	<0.01	FBN2
0.333	<0.01	FAS
0.332	<0.01	COMP
0.327	<0.01	FMOD
0.326	<0.01	CDH2
0.325	<0.01	FUCA1
0.321	<0.01	SGCD
0.314	<0.01	EDIL3
0.314	<0.01	SNTB1
0.312	<0.01	SLIT2
0.308	<0.01	LRRC15
0.307	<0.01	CAPG
0.304	<0.01	LAMA1
0.304	<0.01	CAP2
0.3	<0.01	IGFBP4
0.3	<0.01	MCM7
0.297	<0.01	MMP3
0.297	<0.01	MGP
0.295	<0.01	VCAM1
0.293	<0.01	QSOX1
0.292	<0.01	MATN3
0.29	<0.01	ENO2
0.287	<0.01	NNMT
0.286	<0.01	PCOLCE2
0.278	<0.01	JUN
0.269	<0.01	SGCB

0.269	<0.01	GADD45B
0.268	<0.01	PFN2
0.267	<0.01	ECM1
0.267	<0.01	IL6
0.255	<0.01	VEGFA
0.252	<0.01	SFRP4
0.249	<0.01	MEST
0.248	<0.01	CXCL6
0.246	<0.01	RGS4
0.242	<0.01	FOXC2
0.238	<0.01	SGCG
0.237	<0.01	ELN
0.229	<0.01	PTX3
0.226	<0.01	ABI3BP
0.223	<0.01	CXCL1
0.217	<0.01	IL32
0.214	<0.01	PVR
0.212	<0.01	FBLN5
0.206	<0.01	MSX1
0.19	0.01	SLIT3
0.18	0.02	TIMP1
0.177	0.02	RHOB
0.173	0.02	SERPINE2
0.171	0.02	SPP1
0.164	0.03	CDH6
0.163	0.03	SLC6A8
0.162	0.03	TNFRSF11B
0.162	0.03	ID2
0.146	0.05	CADM1
0.141	0.06	FZD8
0.136	0.07	TGFBR3
0.129	0.09	PLOD3
0.094	0.22	CXCL12
0.08	0.29	MMP1
0.059	0.44	TFPI2
0.059	0.44	SFRP1
0.051	0.51	MATN2
0.032	0.67	MAGEE1
−0.023	0.76	SCG2
−0.035	0.64	APLP1
−0.052	0.5	ANPEP
−0.148	0.05	PRSS2
−0.177	0.02	IGFBP2

Table S2. Clinical and pathological features between *ANXA1* low and high pancreatic cancer in the TCGA cohort.

		**Low (88)**	**High (88)**	***p*-value**
Age	Median	66	65	0.482
	IQR	59–72	56–73	
Race	CA	77	78	1.00
	Others	11	10	
AJCC Stage	I	10	11	0.815
	II	72	73	
	III	2	1	
	IV	1	3	
	Unknown	3	0	
T-category	I	3	4	0.607
	II	14	9	
	III	67	74	
	IV	2	1	
	Unknown	2	0	
N-category	N-	24	25	1.00
	N+	60	62	
	Unknown	4	1	
M-category	M-	30	48	1.00
	M+	1	3	
	Unknown	57	37	
Grade	1	21	10	0.014
	2	47	46	
	3	17	31	
	4	2	0	
	Unknown	1	1	
Tumor site	Head	65	72	0.409
	Body/Tail	16	12	
	Unknown	7	4	
Histology	PDAC	69	76	0.235
	Others	19	12	

*AJCC, The American Joint Committee on Cancer; CA, Caucasian; IQR, interquartile range; PDAC: pancreatic ductal adenocarcinoma.

Table S3. Pancreatic cancer cell lines.

Primary	Metastasis
BXPC3	ASPC1
CAPAN2	CAPAN1
HPAC	CFPAC1
KP2	HPAFII
MIAPACA2	HS766T
PANC0203	HUPT3
PANC0327	HUPT4
PANC0403	KP3
PANC0504	KP4
PANC0813	L33
PANC1005	PATU8988S
PATU8902	PATU8988T
PK45H	PK1
PSN1	PK59
QGP1	SNU410
SW1990	SU8686
	SUIT2
	TCCPAN2
	YAPC

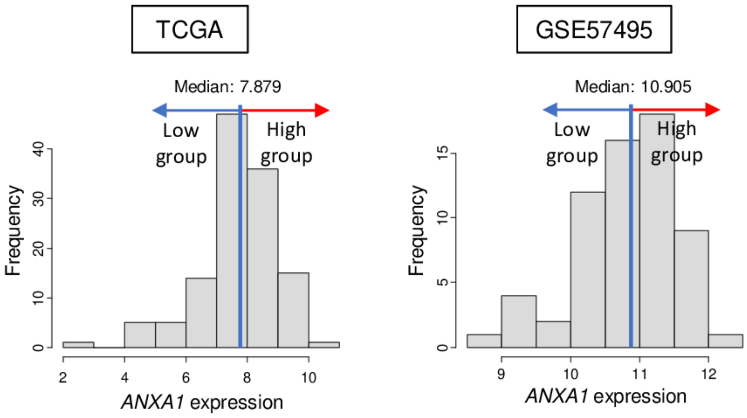

Figure S1. Histogram of *ANXA1* expression in the TCGA and GSE57495 cohorts**.** Median cut-off within each cohort was used to divide into *ANXA1* low and *ANXA1* high groups (*n* = 88, respectively in the TCGA; *n* = 31, and 32, respectively in the GSE57495 cohort).

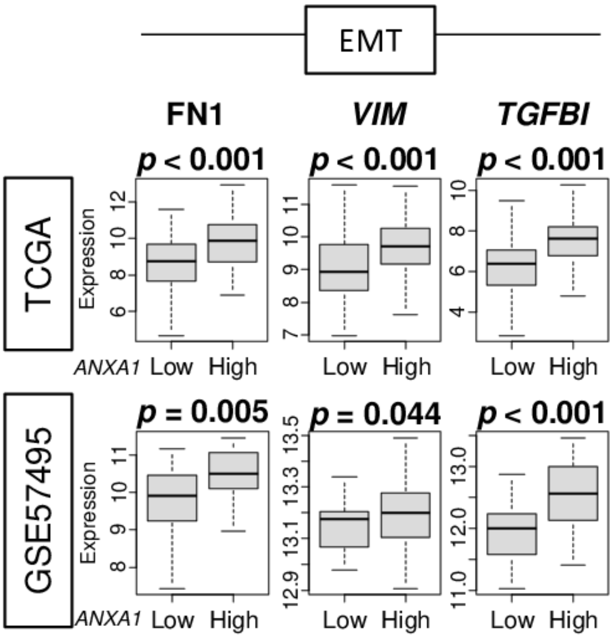

Figure S2. Association of *ANXA1* with epithelial-mesenchymal transition (EMT)-associated gene in the TCGA and GSE57495 cohorts. Boxplots of EMT-associated gene expression; *FN1, VIM,* and *TGFBI,* by low and high *ANXA1* PC. *P*-values were calculated by Mann-Whitney U test. *FN1* (Fibronectin 1), *VIM* (Vimentin), and *TGFBI* (Transforming growth factor, be-ta-induced).
